# Techno-Economic Optimization of Multistage Membrane Processes with Innovative Hollow Fiber Modules for the Production of High-Purity CO_2_ and CH_4_ from Different Sources

**DOI:** 10.1021/acs.iecr.2c01138

**Published:** 2022-06-02

**Authors:** Ricardo Abejón, Clara Casado-Coterillo, Aurora Garea

**Affiliations:** † Departamento de Ingeniería Química, Universidad de Santiago de Chile (USACH), Av. Libertador Bernardo O’Higgins 3363, Estación Central, Santiago 9170019, Chile; ‡ Departamento de Ingenierías Química y Biomolecular, Universidad de Cantabria, Av. Los Castros s/n, Santander 39005, Spain

## Abstract

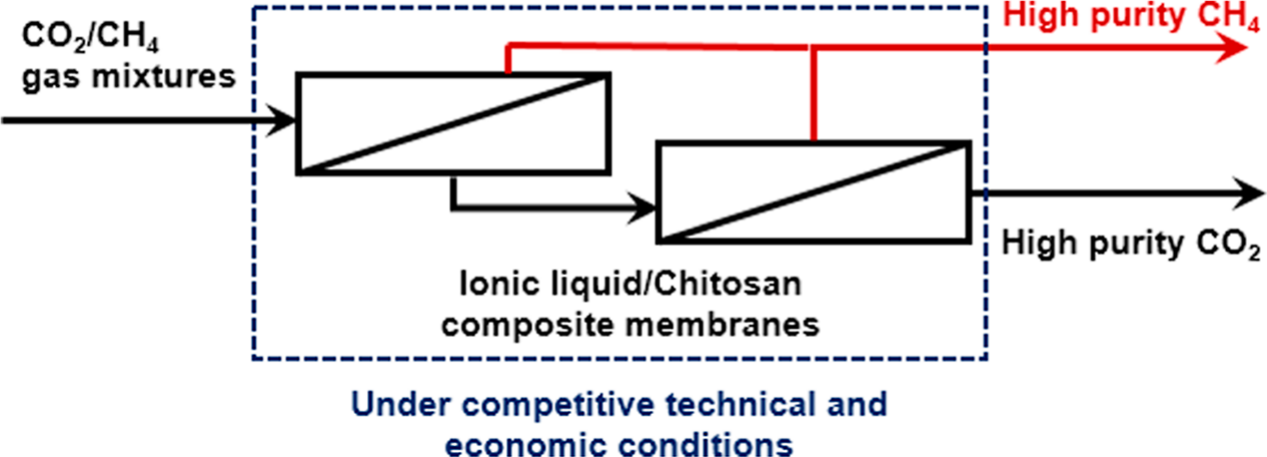

Within the current climate emergency framework and in order to avoid the most severe consequences of global warming, membrane separation processes have become critical for the implementation of carbon capture, storage, and utilization technologies. Mixtures of CO_2_ and CH_4_ are relevant energy resources, and the design of innovative membranes specifically designed to improve their separation is a hot topic. This work investigated the potential of modified polydimethylsiloxane and ionic liquid–chitosan composite membranes for separation of CO_2_ and CH_4_ mixtures from different sources, such as biogas upgrading, natural gas sweetening, or CO_2_ enhanced oil recovery. The techno-economic optimization of multistage processes at a real industrial scale was carried out, paying special attention to the identification of the optimal configuration of the hollow fiber modules and the selection of the best membrane scheme. The results demonstrated that a high initial content of CH_4_ in the feed stream (like in the case of natural gas sweetening) might imply a great challenge for the separation performance, where only membranes with exceptional selectivity might achieve the requirements in a two-stage process. The effective lifetime of the membranes is a key parameter for the successful implementation of innovative membranes in order to avoid severe economic penalties due to excessively frequent membrane replacement. The scale of the process had a great influence on the economic competitiveness of the process, but large-scale installations can operate under competitive conditions with total costs below 0.050 US$ per m^3^ STP of treated feed gas.

## 1 Introduction

The climate change due to global warming is the most demanding environmental challenge to be faced by our society. The continuous increase of greenhouse gases emissions causes great concern, and environmental consequences have become a priority.^1−3^ Carbon dioxide (CO_2_) is the most relevant greenhouse gas, and consequently, carbon capture, storage, and utilization technologies have been widely developed during the last decade.^4−8^ All these technologies require adequate gas separation processes, and membranes play a very important role in this scenario.^9−11^ Membranes allow the selective permeation of gas through them, and their selectivity to the different gases present in a mixture is intimately related to the nature of the membrane material.

The development of membranes specifically designed for the separation of carbon dioxide/methane (CO_2_/CH_4_) mixtures has gained importance,^12−14^ and various examples of applications related to energy resources that require this separation can be mentioned. For instance, natural gas consists primarily of CH_4_, but commonly contains significant amounts of CO_2_ as impurity. Because of the lack of heating value and the corrosive nature of CO_2_ in the presence of humidity, natural gas must be subject to a sweetening process, which removes CO_2_ and other acid gases present as impurities in order to make it suitable for commercial applications.^15−17^ In addition, the biogas resulting from the anaerobic digestion of biomass waste is mainly a mixture of CH_4_ and CO_2_.^18,19^ Biogas can be upgraded to bio-methane, which can replace traditional natural gas, but once again, removal of CO_2_ is required. Another example that can be mentioned is the gas mixture recovered after CO_2_ enhanced oil recovery. This oil displacement technology can increase the crude recovery by 25% and is widely used in many oil fields.^20,21^ When CO_2_ is injected, crude oil is greatly swelled, reducing its viscosity and interfacial tension and thereby improving the fluidity of crude oil. However, when CO_2_ is used to enhance oil recovery, the large amount of oilfield-associated gas (mainly CH_4_) gets diluted. Due to the resulting high CO_2_ concentration, the oilfield-associated gas cannot be directly burned, while reinjecting the associated gas directly to enhance oil recovery does not meet the basic requirement that the minimum miscibility pressure must be lower than the that of the reservoir.^22^ Therefore, the separation of CH_4_ and CO_2_ is required to exploit adequately both resources.

In the last decade, significant progress has been observed in membrane science, as new classes of polymers have been developed with improved performance for CO_2_ separation, including the CO_2_/CH_4_ gas pair. Many different types of membranes, such as polymeric membranes, metal–organic frameworks, mixed-matrix membranes, carbon membranes, or silica and zeolite membranes, have been prepared for the separation of CO_2_ and CH_4_.^23−27^ This research group has previous experience in the preparation of innovative membranes for CO_2_/CH_4_ mixture separation. On the one hand, improved membranes have been synthetized by addition of ionic liquids.^28^ On the other hand, chemical modification of commercially available membranes has been tested.^29^ Despite these efforts to improve the performance of commercially available membranes and reduce the environmental impacts of innovative membranes by consideration of environmentally friendly biopolymers as membrane materials, initial process engineering tasks have demonstrated that the separation process performance in terms of purity and recovery would not be enough to obtain streams that fulfill the requirements imposed to CO_2_ and CH_4_ direct valorization in a single membrane stage. Consequently, further efforts must be taken into consideration to design more complex multistage separation processes that can achieve the imposed requirements.^30^ Sometimes, these multistage processes require the design of complex membrane networks, with multiple membrane units in series under multistage and multistep configurations, that require recycle streams. Examples of even 7 membrane stages have been proposed,^31^ including auxiliary equipment such as mixers, separators, compressors, vacuum pumps, or heat exchangers. Such complex processes can be simplified when membranes with exceptional performance are implemented. Therefore, the evaluation of the technical viability and economic competitiveness of these innovative membranes with enhanced performance for the separation of CO_2_/CH_4_ mixtures in simple multistage schemes is essential in order to compare them with other alternatives.^32−34^

The aim of this work is the techno-economic optimization of a simple multistage process based on innovative membranes for the separation of CO_2_ and CH_4_ mixtures from different sources, such as biogas upgrading, natural gas sweetening, or CO_2_ enhanced oil recovery, at an industrial scale. A non-commercial ionic liquid–chitosan composite membrane, which can be considered a greener option when compared to other polymeric membranes, is compared with a commercial polydimethylsiloxane membrane (with and without further chemical modification) in order to demonstrate its competitiveness in both technical and economic terms. The optimization includes the configuration of the hollow fiber membrane modules and the selection of the best membrane scheme in simple multistage processes (just two stages without recycle streams) to complete the assessment of the optimal operation conditions according to technical and economic criteria.

## 2 Design, Simulation, and Optimization of Multistage Systems for Membrane-Based Separation of CO_2_: State of the Art

The modeling and simulation of gas permeation membrane processes are reported in the literature for different applications, such as recovery of carbon dioxide from large emission sources (post-combustion capture), removal of nitrogen from air for oxy-combustion in power plants, natural gas sweetening, and treatment of biogas. The simulation software tools and programming languages employed for the simulation of these processes include CHEMCAD, Aspen Custom Modeler, Aspen Plus, Aspen HYSYS, PROII, or MATLAB (Table 1). Most of these representative studies are focused on simulating the performance of the membrane separation processes, paying special attention to sensitivity analysis in order to identify and represent the importance of design and operation variables (mainly temperature, pressure, feed composition, membrane characteristic permeability and selectivity, and number of stages) on the purity of the product streams, the process yield, and the economic costs (emphasizing the energy requirements in separation processes and compression or vacuum operations).

**Table 1:** Main Commercial Simulation and Mathematical Modeling Tools Applied to Gas Separation Processes by Membranes

software tool	application	references
PRO/II	flue gas (coal power plant)	(35−37)
CHEMCAD	flue gas (coal/natural gas power plants)	(38, 39)
CHEMCAD	syngas (IGCC plant)	(40)
CHEMCAD	flue gas (cement)/blast furnace gas	(41)
Excel	syngas (IGCC plant)	(42)
COMSOL	flue gas (coal power plant)	(43)
MATLAB	natural gas upgrading	(44)
MATLAB	flue gas (power plant)	(45)
MATLAB	flue gas (power plant)	(46)
MATLAB	flue gas (coal power plant)	(47)
MATLAB	flue gas (coal power plant)	(48)
MATLAB	natural gas upgrading	(49)
Aspen Custom Modeler	flue gas (coal power plant)	(50)
Aspen Custom Modeler and Excel	flue gas (LNG power plant)	(51)
Aspen Custom Modeler and Aspen Plus	flue gas (coal power plant)	(52)
Aspen Plus and JACOBIAN	oxy-combustion	(53)
Aspen Plus and EbsilonProfessional	syngas (IGCC plant)	(54)
Aspen Plus and FORTRAN	flue gas (coal power plant)/oxy-combustion/syngas (IGCC plant)	(55, 56)
Aspen Plus and FORTRAN	syngas (IGCC plant)	(57)
Aspen Plus and FORTRAN	syngas (IGCC plant)	(58)
Aspen Plus and MEMSIC	natural gas upgrading	(59)
Aspen Plus and MEMSIC	blast furnace gas	(60)
Aspen Plus and MEMSIC	direct air capture	(61)
Aspen HYSYS	flue gas (coal power plant)	(62)
Aspen HYSYS	natural gas upgrading	(63)
Aspen HYSYS	natural gas upgrading	(64, 65)
Aspen HYSYS and CAPCOST	blast furnace gas	(66)
Aspen HYSYS and Visual Basic	natural gas upgrading	(67)
Aspen HYSYS and ASPEN Icarus	syngas (IGCC plant)	(68)
Aspen HYSYS and MemCal	natural gas upgrading	(69)
Aspen HYSYS, ChemBrane and CAPCOST	flue gas (coal power plant)	(70)
Aspen HYSYS, ChemBrane and CAPCOST	biogas	(71)

Several studies have indicated that a single stage membrane process cannot achieve CO_2_ capture values above 90% in flue gases and produce a high purity CO_2_ permeate stream, regardless of the type of membrane used due to the limited practical transmembrane pressure ratio and membrane selectivity.^72^ Taking into account this limitation, the treatment of flue gases requires the design of multistage membrane processes for the recovery of CO_2_ achieving purity and recovery acceptable values. The design of multistage membrane systems allows the proposal of various configurations to meet the defined separation objectives. Nevertheless, for direct comparison of these different designs, the definition of common performance indicators is required and the total economic costs of 1 ton of CO_2_ captured can be selected as the functional unit. Thus, in the literature, there are reported cost values in the range of 20–90 US$/ton CO_2_, which consider the sum of the costs due to CO_2_ separation (equipment and operation) and subsequent liquefaction and compression (a reference value may be 140 bar).

The driving force of membrane-based gas separation is the partial pressure difference between the feed and the permeate side. Due to the limitations of the operating conditions of the post-combustion capture (CO_2_ concentrations of the order of 13–15% at atmospheric pressure), the partial pressure difference of CO_2_ must be modified to increase the driving force. On the one hand, the feed side can use a compressor to increase the pressure, and the CO_2_ concentration can be increased by recirculation of an enriched CO_2_ stream. On the other hand, on the permeate side, the pressure can be decreased using a vacuum pump or the concentration of CO_2_ can be reduced using a sweep gas.

**Figure 1:**
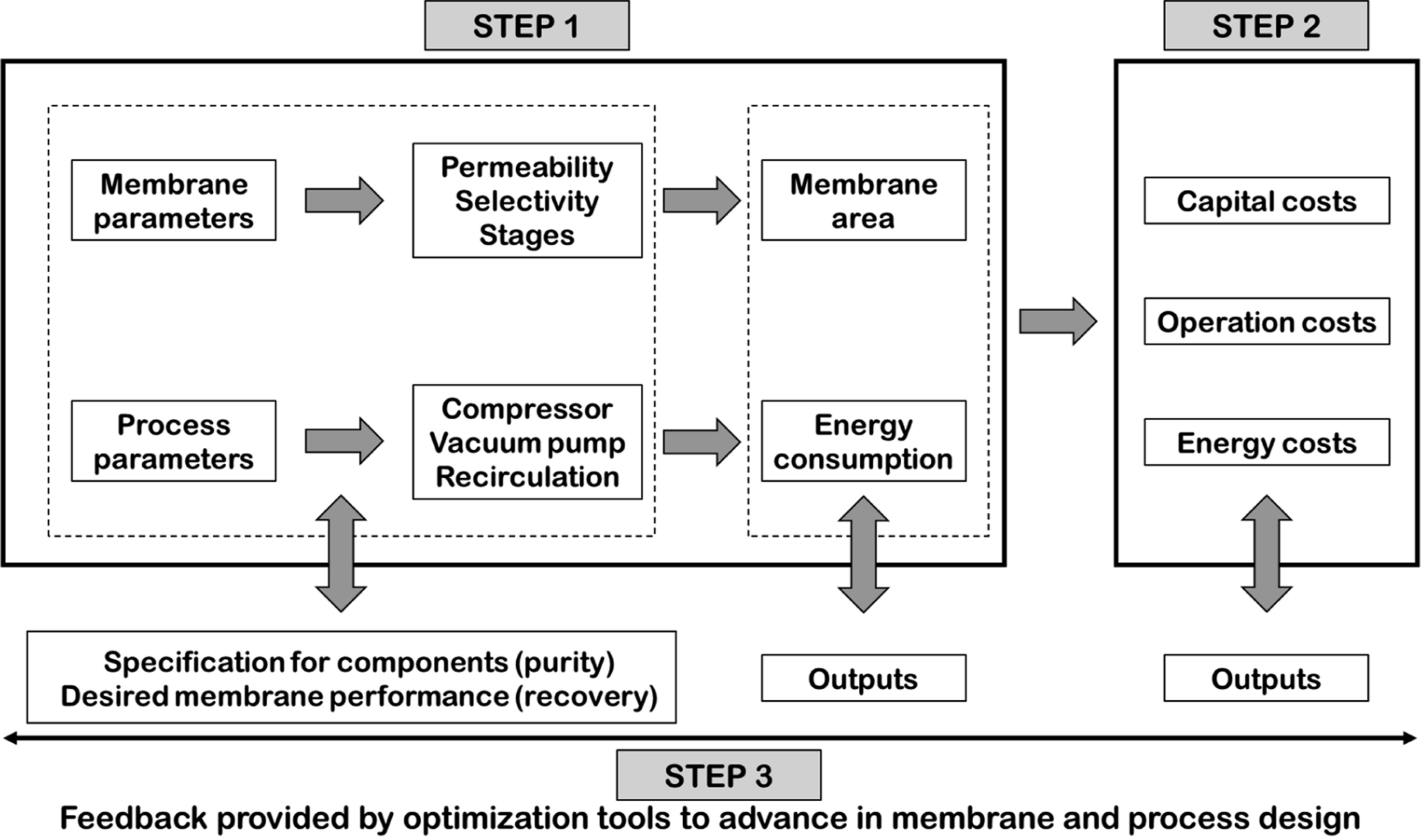
Schematic diagram of the research strategy for the techno-economical optimization of a membrane separation process for CO_2_ purification.

The application of compressors and vacuum pumps implies consumption of electrical energy, which causes an energy penalty in the existing power plants. For this reason, a key aspect in multistage designs for CO_2_ capture is the simultaneous minimization of both energy consumption and total capture costs. A diagram of the research strategy is illustrated in Figure 1, which summarizes these objectives. The simulation process can be divided into two tasks.^36^ First, the influence of the membrane parameters on the membrane area required to achieve the specified goal is analyzed. This step can be combined with the evaluation of various process parameters, system components, and configurations of the membrane units. The values of membrane area and energy consumption are the two outcomes of the results presented in this task. Second, the effect of the variation of these membrane and process parameters on the economic cost is analyzed, especially the correlation between the membrane parameters (selectivity and permeability) and the capture performance (capital, operation, and energy costs). The corresponding simulation tasks based on this approach investigated the performance of a two-stage cascaded membrane process to replace the conventional process using amines in absorption columns.^35,37^ A reference power plant was chosen for this purpose: a 600 MW capacity electric coal-fired power generation plant installed in North Rhine-Westphalia, where the membrane separation process was implemented after the treatment systems required for other acid gas and particle elimination (SCR-DeNOx, E-filter, and FGD) and prior to the cooling towers. The results indicated that the process of capture of CO_2_ by membranes can involve lower energy costs than the conventional process of absorption with amines, and this difference is more favorable when percentages of capture of CO_2_ lower than 90% are considered.

The requirement of multiple membrane stages for effective gas separation processes implies the design of membrane networks, which allow the installation of multiple membrane units in series, defining multi-stage and multi-step configurations where recycle streams can be connected to different points of the network. The selection of the optimal membrane network for a determined application is not a simple task. Synthesis schemes in the form of superstructures are applied in various areas of process development, such as power and utility systems, water reuse networks, wastewater management, chemical reactors, separation and reactive separation trains, and product design. This approach is useful to the case of membrane networks, and the problem of membrane cascade design has been considered for the generation of the configurations and their optimization using a superstructure concept, with mass and energy integration.^73^ While the case of membrane networks presents similarities to general separation schemes, they also pose new challenges. Consideration of pressure warrants additional consideration in the design approach. Nevertheless, the equations that represent the configurations of membrane networks and interconnections in superstructures can be solved using different programs or commercial packages of mathematical programming for optimization such as GAMS (GAMS Development Corporation), gPROMS (Process Systems Enterprise Limited), LINGO, and Lindo API (Lindo Systems Inc.) or AMPL (AMPL Optimization Inc.). All these tools provide solver libraries for different optimization problems that require handling large systems of equations with discrete and continuous variables, currently applying global optimization techniques and multi-objective optimization algorithms.

The scheme of a generic superstructure for membrane separations can include different elements in the process design: membrane units, mixers, separators, compressors, vacuum pumps, heat exchangers, and their possible interconnections.^74^ The number of stages to be considered in the design of the process can be worked out as fixed parameters or as discrete variables that require solving the proposed model with mixed integer nonlinear programming (MINLP) algorithms. MINLP refers to optimization problems with continuous and discrete variables and nonlinear functions in the objective. It is important to note that the consideration of hybrid processes is also being addressed in the designs of the CO_2_ capture processes, with different types of membranes for the different separation units, or even combination with other separation processes.^48,60^ In addition, the boundaries of the system have been expanded to include both the capture and the stages needed for conditioning and transport CO_2_. This way, the influence of all the stages of the capture process in a global model of carbon capture and storage or carbon capture and utilization can be further investigated.^75^ The solutions proposed by these advanced configurations can further reduce the total costs of the CO_2_ capture and achieve values below 22 US$/ton CO_2_. Table 2 compiles some of the most recent research works regarding the optimization of membrane-based separation processes focused on CO_2_ considering multiple-unit systems and superstructures.

**Table 2:** Some Recent Research Works Focused on the Optimization of Membrane Separation Processes: Multistage Designs and Superstructures

application	feed composition	maximal number of stages	references
post-combustion CO_2_ capture (flue gas)	CO_2_/N_2_	3	(76)
		4	(77)
		4	(74)
		6	(78)
		3	(75)
		3	(79)
		2	(48)
		4	(80)
		2	(81)
		7	(31)
		2	(82)
		3	(52)
natural gas upgrading	CO_2_/CH_4_	4	(44)
		3	(83)
		4	(84)
		3	(69)
		5	(85)
biogas purification	CO_2_/CH_4_	3	(86)
		3	(87)
		3	(88)
		3	(89)
		3	(90)
blast furnace gas	CO_2_/CO/N_2_/H_2_	3	(91, 92)
pre-combustion CO_2_ capture (syngas)	CO_2_/H_2_	3	(42)

## 3 Modeling

**Figure 2:**
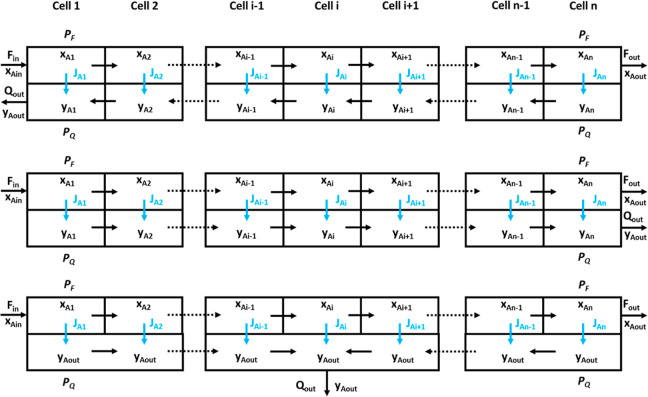
Scheme of the module configurations considered in this work: counter-flow (up), co-low (middle), and cross-flow (down).

The modeling of membrane separation of gas mixtures in hollow fiber modules has been performed using different approaches. In this work, the model previously presented in a preceding paper from this research group has been applied.^30^ The model had been previously validated with experimental data. The hollow fiber is divided into a series of *n* perfectly mixed cells in the axial direction and mass balances are enforced in each section. This procedure is formally equivalent to using first order finite differences to develop a set of coupled difference equations from the differential mass balances. According to previous results, the number of cells was equal to 100 since this value gets an adequate balance between precision and calculation load. The bore-side feed countercurrent flow arrangement was the module configuration selected in that case, and the mathematical model was applied for this specific configuration. Since alternative configurations, such as bore-side feed co-current flow and lumen side permeate cross-flow (Figure 2), have been considered in this case as well, the model has been adapted to be applied to these other configurations.

The model is based on the corresponding global and component material balances to the module and the *n* stages, coupled with the characterization of gas transport through the membrane and the definition of module design and performance parameters. Only the equations for the most permeable component A are described here in detail, as the equations of the least permeable compound B are equivalent. The co-current flow configuration is defined by the following equations:

Material balances on the module are
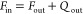
1
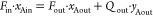
2
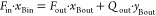
3

Material balances on the cell (global, on the tube side, and on the shell side)
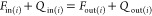
4
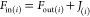
5
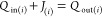
6

Flow across the membrane is
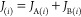
7
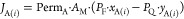
8
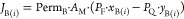
9

Cell continuity is
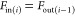
10
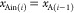
11
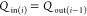
12
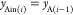
13

Relationship between the individual and total flows (definition of molar fractions) is
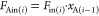
14
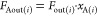
15
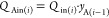
16
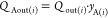
17

Membrane transport properties are
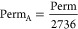
18
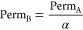
19

Definition of module design and performance parameters is
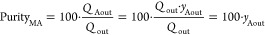
20
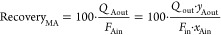
21
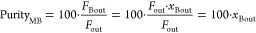
22
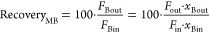
23
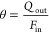
24
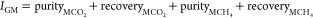
25

In the case of cross-flow configuration, the same model can be used just replacing the equations related to material balance on the shell side of the cell (eq 6) and the definition of the flow across the membranes (eqs 8 and 9):

Material balances on the cell (on the shell side) are given by
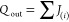
6b

Flow across the membrane is given by
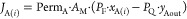
8b
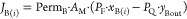
9b

**Figure 3:**
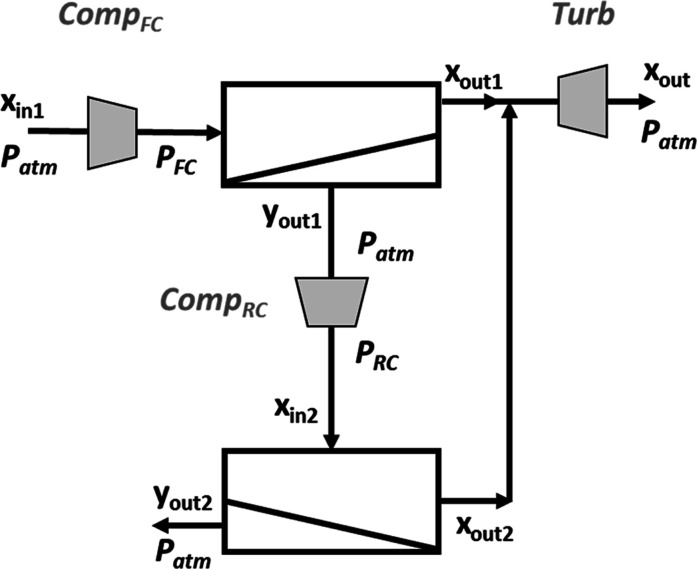
Schematic representation of the 2-stage process considered in this work.

The process configuration required for high effective separation of CO_2_ and CH_4_ mixtures, which must be able to attain simultaneously high recovery and purity values for both gases, must include more than one membrane unit in order to achieve the imposed restrictions successfully. Different membrane network superstructures can be defined and screened by an optimization technique to identify the optimal process configuration, and several examples of this approach have been reported.^60,74,76,82,93^ This approach can derive in the design of quite complex systems, with a high number of stages, such as processes with more than five stages^31,78^ or the presence of several recirculation and by-pass streams.^85,94,95^ Nevertheless, the application of innovative membranes with enhanced separation characteristics allows the design of much more simple process configurations, with just two stages with permeate connection in series and without recirculation or by-pass streams.^96^ The schematic diagram of the membrane system configuration developed in this work is shown in Figure 3. This connection between the two module stages is represented by the corresponding continuity equations:

Stream continuity
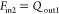
26
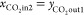
27
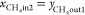
28

The definition of the process performance parameters was equivalent to the ones defined for just a membrane module.

Definition of process performance parameters is given as
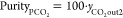
29
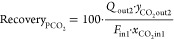
30
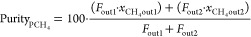
31
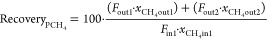
32
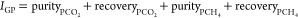
33

The economic evaluation model was developed taking into account the mathematical cost estimation originally proposed for the upgrading of low-quality natural gas with H_2_S and CO_2_ by means of selective polymeric membranes.^97^ However, it has been properly adapted for its application to the cases considered in this work by incorporation of the system for energy recovery from the high-pressure outlet stream and the cooling costs of multistage compressors under non-isothermal conditions.^84^

The total costs of the separation process take into account the capital related costs (CC), the variable operation and maintenance cost (OC), and the cost of methane losses in the permeate stream (LSC):

Total costs are given by
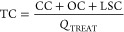
34
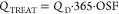
35
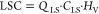
36
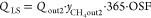
37

The capital costs are mainly based on the fixed costs (FC) due to the investment in equipment, such as membrane modules, compressors, heat exchangers, and turbine (all these costs have been referenced to 2018 prices by the corresponding CEPCI cost indexes), but aspects such as project contingency and start-up costs are considered too:

Capital costs are given by
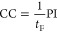
38
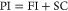
39
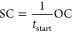
40
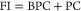
41
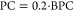
42
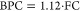
43

Fixed costs are given by
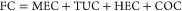
44
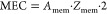
45
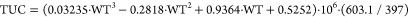
46
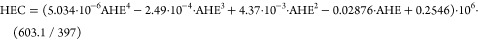
47
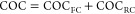
48
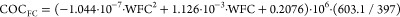
49
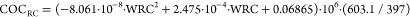
50

The operation costs are essentially based on the consumption of the corresponding resources: utilities (UC), membrane replacement (MRC), and labor (LC), except for the case of maintenance costs (MC) and insurance costs (IC), which are a function of the total capital costs.

Operation costs are given by
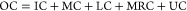
51
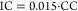
52
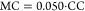
53
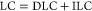
54
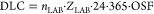
55
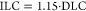
56
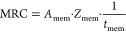
57
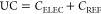
58
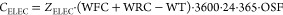
59
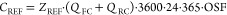
60

Finally, additional equations are required to consider the performance of the compressors, heat exchangers, and turbine.

The performance of feed compressor and heat exchanger are given by
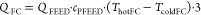
61
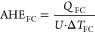
62
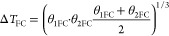
63
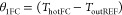
64
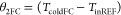
65
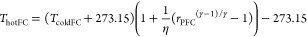
66
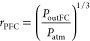
67
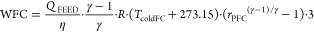
68

The performance of second stage compressor and heat exchanger is given by
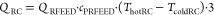
69
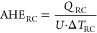
70
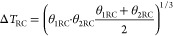
71
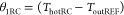
72
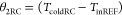
73
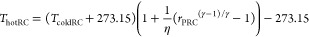
74
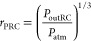
75
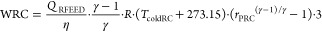
76

Total heat exchanger area is given by
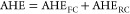
77

The performance of turbine is given by
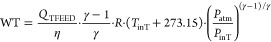
78

The pressures on the feed side of both stages are independent, and compressors fed at atmospheric pressure are employed for the pressurization until the optimal values. The pressure drop along the axial direction of the membrane module is assumed to be negligible.^81^ The compressors are modeled as three-stage compressors (compression ratio is assumed to be the same in each stage), and heat exchangers are used after feed compression (the temperature of the refrigeration water entering the heat exchangers is 5 °C, while the outlet temperature increased until 15 °C) to cool gas streams down to the membrane optimal operation temperature (30 °C). Nevertheless, the operation temperatures are assumed to have a negligible effect on membrane performance.^84^

## 4 Case Studies and Optimization Methodology

Three different case studies were defined to take into account various representative examples of the separation CO_2_/CH_4_ mixtures with industrial interest. First, a biogas upgrading installation was proposed. In this case, the composition of the CO_2_/CH_4_ mixtures was 35:65% and the feed stream flowrate was 200 m^3^ STP/h, which can be considered a small-scale plant.^98−102^ The second case study covered the design of an installation for natural gas sweetening, which was characterized by a higher content of CH_4_ in the mixture (10:90% composition) and a much higher gas volume to be treated since the feed stream flowrate was 6000 m^3^ STP/h.^103^ Finally, the design of an installation for enhanced oil recovery was carried out. In this case, the gas mixture was richer in CO_2_ (60:40% composition) and the feed stream flowrate was 200 m^3^ STP/h.^22^ In all cases, the corresponding feed streams were considered binary mixtures, although it is known that biogas and wellhead natural gas are more complex multicomponent mixtures. Nevertheless, this simplification is common when modeling membrane gas separations.^22,34^ The product requirements for purity and recovery were similar for the three case studies: on the one hand, the recovery of CO_2_ must be at least 90% with 90% purity, while, on the other hand, the recovery of CH_4_ must be not lower than 95%, and the concentration in the product stream must be at least 98%.^98,104^ The separation processes were designed in order to maintain the stage cut values between 0.05 and 0.95, while the maximal applied pressure in the membrane modules was limited to 20 bar.

Three different membranes, called PDMS (commercial polydimethylsiloxane membrane), PDMS_t_ (modified commercial polydimethylsiloxane membrane), and IL2 (non-commercial ionic liquid–chitosan composite membrane), were investigated and their permeability and selectivity properties are compiled in Table S1,^30^ with the rest of the parameters required by the model. These permeability and selectivity values of the membranes were assumed to be constant (independent of temperature) since permeation through the membrane can be considered an isenthalpic process, which implies only a small temperature change that can be neglected. Constant membrane permeability and selectivity were considered in all cases, including the lowest concentration values, which were close to 1%. Besides, some authors have demonstrated that low concentration ranges can improve the performance of the membranes^105^ so the constant values defined in this work could be considered a worst-case conservative scenario for these low concentrations.

In mathematical terms, the optimization problem can be expressed as follows:
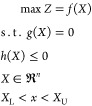
being *Z* the techno-economic objectives, *x* is the vector of decision variables (applied pressures, module stage cuts), *g* is the vector of equality constraint functions (mass balances, membrane transport equations, equipment and system performance equations, and economic considerations), and *h* is the vector of inequality constraint functions (product requirements based on the purity and recovery limits for each gas and limits to the operation conditions). GAMS software (34.3 version) was employed as the optimization tool to solve the developed nonlinear programming model using the CONOPT solver. The General algebraic modeling system (GAMS) is a high-level modeling system for mathematical programming and optimization. It consists of a language compiler and a stable of integrated high-performance solvers.^106^

## 5 Results and Discussion

### 5.1 Optimization of the Module Configuration

In order to characterize the performance of the different membrane modules under the three proposed configurations (counter-flow, co-flow, and cross-flow), the optimization of the process to treat 1 m^3^ STP/h of biogas (35:65% mixture), maximizing the global performance of the module (*I*_GM_), was carried out. The results are compiled in Table 3.

**Table 3:** Performance of the Membrane Modules under the Different Configurations Considered in This Work

membrane	module configuration	purity_MCO_2__	recovery_MCO_2__	purity_MCH_4__	recovery_MCH_4__	*I*_GM_	stage cut θ	area (m^2^)
PDMS	counter-flow	49.1	84.8	86.6	52.7	273.3	0.604	0.681
	co-flow	49.3	81.8	84.8	54.6	270.6	0.581	0.655
	cross-flow	49.3	82.4	85.2	54.4	271.4	0.585	0.658
PDMS_t_	counter-flow	68.1	90.2	93.6	77.2	329.1	0.464	3.696
	co-flow	68.6	85.8	91.2	78.8	324.5	0.438	3.450
	cross-flow	68.7	86.4	91.5	78.8	325.4	0.440	3.461
IL2	counter-flow	86.2	94.8	97.0	91.9	369.9	0.385	4.562
	co-flow	87.8	89.1	94.1	93.4	364.3	0.355	3.785
	cross-flow	87.8	89.3	94.2	93.3	364.7	0.356	3.790

As expected, the results revealed that the counter-flow configuration attained the highest *I*_GM_ values for all the tested membranes,^107,108^ while the global performance of the cross-flow configuration was slightly higher than that of the co-flow configuration. Specifically, the *I*_GM_ values obtained for the counter-flow configuration were 1.0–1.5 and 0.7–1.4% higher than those of the co-flow and cross-flow, respectively (the highest margin of improvement corresponded to the IL2 membrane, while the PDMS membrane showed the lowest). Although the global performance was better applying the counter-flow configuration, this better result was based on the higher values of CO_2_ recovery and CH_4_ purity as a consequence of the more stable gradients throughout the module that were maintained in the counter-flow mode, which resulted in increased gas permeation but the cross-flow configuration attained the highest values of CH_4_ recovery and CO_2_ purity. The highest transport of CO_2_ from the feed stream to the permeate stream in the counter-flow configuration resulted in the highest stage cut values and required more membrane area than the other configurations. For instance, the percentages of the additional membrane area required under counter-flow conditions when compared to the co-flow alternative (characterized by the lowest membrane area) summed up 4, 7, and 20% for PDMS, PDMS_t_, and IL2 membranes, respectively.

**Figure 4:**
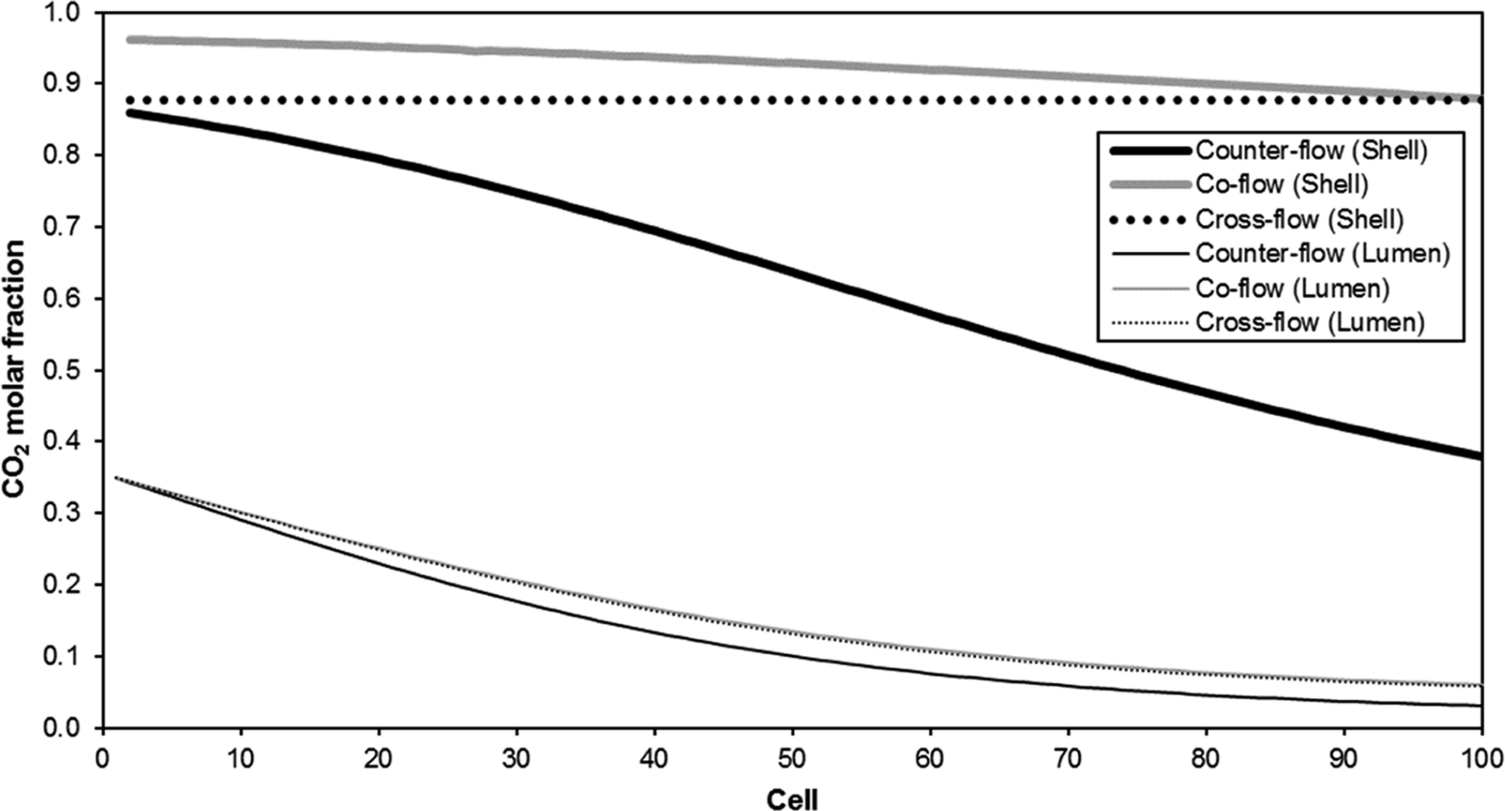
Profiles of the CO_2_ molar fraction of the shell and lumen sides of the modules under the different configurations.

These facts were clearly observed in detail when the profiles of the gas concentration throughout the modules were analyzed (Figure 4). The evolution of the CO_2_ concentration in the lumen side exhibited the enhanced transport of CO_2_ when counter-flow confirmation was applied, allowing lower CO_2_ concentration when compared to the alternative configurations. The profiles of the shell side are quite different as a direct consequence of the different configurations: the cross-flow configuration exhibited a constant concentration value, the co-flow attained the maximal CO_2_ concentration in the inlet (left side of the graph) to reduce this value throughout the module due to increased CH_4_ permeation as it was enriched in the lumen side, while the counter-flow showed the highest concentration variation from the inlet (right side of the graph) to the outlet (left side of the graph). According to the obtained results, the counterflow configuration was selected to be implemented in the membrane modules since it provided the maximal CO_2_ transport through the membranes.

### 5.2 Selection of an Optimal Membrane Scheme

As demonstrated by the results compiled in Table 3, the design of a membrane separation process based on just a single stage is not realistic even when membranes with improved characteristics are employed because the purity and recovery objectives cannot be achieved. Therefore, the design of processes including at least two stages becomes mandatory. In this work, the optimization of the process to treat 1 m^3^ STP/h of biogas (35:65% mixture), maximizing the global performance of the process (*I*_GP_), was carried out for all the combinations of the evaluated membranes (under cross-flow conditions) in both stages (Table 4).

**Table 4:** Performance of the 2-Stage Process under Different Membrane Schemes

membrane scheme										
stage 1	stage 2	purity_PCO_2__	recovery_PCO_2__	purity_PCH_4__	recovery_PCH_4__	*I*_GP_	stage cut θ_1_	area 1 (m^2^)	stage cut θ_2_	area 2 (m^2^)
PDMS	PDMS	51.3	93.9	94.1	52.0	291.3	0.790	0.948	0.811	0.707
PDMS	PDMS_t_	71.3	92.6	95.2	79.9	338.9	0.806	0.973	0.564	3.353
PDMS	IL2	88.0	95.2	97.2	92.9	373.4	0.857	1.053	0.442	3.987
PDMS_t_	PDMS	70.9	92.6	95.3	79.6	338.4	0.518	4.514	0.882	0.401
PDMS_t_	PDMS_t_	83.3	93.9	96.4	89.8	363.4	0.551	5.059	0.716	2.008
PDMS_t_	IL2	93.2	96.7	98.2	96.2	384.2	0.600	5.970	0.605	2.399
IL2	PDMS	87.6	95.1	97.2	92.8	372.7	0.400	5.285	0.950	0.260
IL2	PDMS_t_	92.4	96.6	98.2	95.8	382.9	0.418	6.306	0.875	1.238
IL2	IL2	96.9	98.5	99.2	98.3	392.9	0.446	8.063	0.798	1.339

The differences among the different membrane pairs were obvious, with great performance when the most selective IL2 membrane was included, especially for the CO_2_ purity and CH_4_ recovery values, which were low when the PDMS membrane was employed. In fact, only three pairs of membranes were able to fulfil the purity and recovery limits imposed: PDMS_t_/IL2, IL2/PDMS_t_, and IL2/IL2. Only these combinations were able to achieve *I*_GM_ values above 380, CO_2_ purity values above 90%, and CH_4_ recoveries about 95%. The selection of the most selective IL2 membrane in both stages attained the most effective separation (*I*_GM_ value equal to 392.9), but the combination of IL2 and PDMS_t_ (indistinctly of the order) also resulted effective for the separation of the biogas mixture. According to Ding, the high pressure ratio of the process (20) implied that the process worked in the membrane selectivity-limited region.^109^ Under these circumstances, the membrane process is significantly benefited from the availability of a high selectivity membrane such as IL2, and the order of the different membranes when IL2 was combined with PDMS_t_ is not greatly significant: the combination PDMS_t_/IL2 just achieved a slightly higher *I*_GP_ value of 384.2 than the IL2/PDMS_t_ combination (*I*_GP_ value of 382.9, which is less than 0.4% reduction when compared to the alternative pair PDMS_t_/IL2). Nevertheless, when both combinations of IL2 and PDMS_t_ were tested with other feed streams (e.g. the oil enhanced recovery mixture 60:40%), they failed and the imposed purity and recovery restrictions were not attained. Consequently, only the designs with both stages implementing the IL2 membrane were considered for complete techno-economic optimization of the real scale processes.

### 5.3 Techno-Economic Optimization

Three different membranes, called PDMS (commercial polydimethylsiloxane membrane), PDMS_t_ (modified commercial polydimethylsiloxane membrane), and IL2 (non-commercial ionic liquid–chitosan composite membrane), were selected for this study. The 5.1 and 5.2 sections covered the performance and the process optimization results, corresponding to the PDMS membrane as a commercial reference membrane in both stages of the proposed process and in hybrid configurations that combined PDMS commercial with the other non-commercial membranes (Tables 3 and 4) in order to fulfill the purity and recovery limits imposed. These membranes were selected because of the different permselectivity performance and stability in the process evaluated so far at the laboratory scale. The optimization of the two-stage process with the IL2 membrane in both stages for all the case studies (biogas, natural gas, and enhanced oil recovery) was carried out considering the technical optimal solution (maximal *I*_GP_ value) and the economic optimal solution (minimal total costs). The main results are compiled in Table 5. The analysis of the results revealed that the purity of CH_4_ was the restriction that limited the economic optimization of the process treating biogas and enhanced oil recovery, while in the case of the natural gas, the limiting restriction was the recovery of CO_2_. Besides, during the technical optimization of the natural gas process, the purity of CO_2_ acted as limiting restriction, while the other two case studies were not subject to limiting restrictions (all the imposed purity and recovery values were surpassed). Therefore, the high initial content of CH_4_ in the feed stream (10:90%) might imply a great challenge for the separation performance of the process, and only membranes with enough selectivity can be implemented for this purpose. When the total costs of the different processes were compared, the case of natural gas presented the lowest costs (0.048 US$/m^3^ STP), with very similar costs for the other two cases (0.288 and 0.285 US$/m^3^ STP for biogas and enhanced oil recovery, respectively).

**Table 5:** Technical and Economic Optimization of the Different Case Studies

	biogas		natural gas		oil recovery	
	technical optimization	economic optimization	technical optimization	economic optimization	technical optimization	economic optimization
feed stream (m^3^/h)	200	200	6000	6000	200	200
purity CO_2_ (%)	96.9	97.8	90.0	91.7	98.6	98.9
recovery CO_2_ (%)	98.5	96.3	94.5	90.0	99.4	98.7
purity CH_4_ (%)	99.2	98.0	99.4	98.9	99.0	98.0
recovery CH_4_ (%)	98.3	98.8	98.8	99.1	97.9	98.4
stage cut θ_1_	0.446	0.404	0.214	0.172	0.664	0.642
stage cut θ_2_	0.798	0.852	0.490	0.573	0.911	0.931
membrane area 1 (m^2^)	1613	1102	54 883	37 788	1186	920
membrane area 2 (m^2^)	268	212	5677	4571	300	266
*P*_FC_ (atm)	20	20	20	20	20	20
*P*_RC_ (atm)	20	20	20	20	20	20
total cost (US$/m^3^)	0.302	0.288	0.062	0.048	0.293	0.285

**Figure 5:**
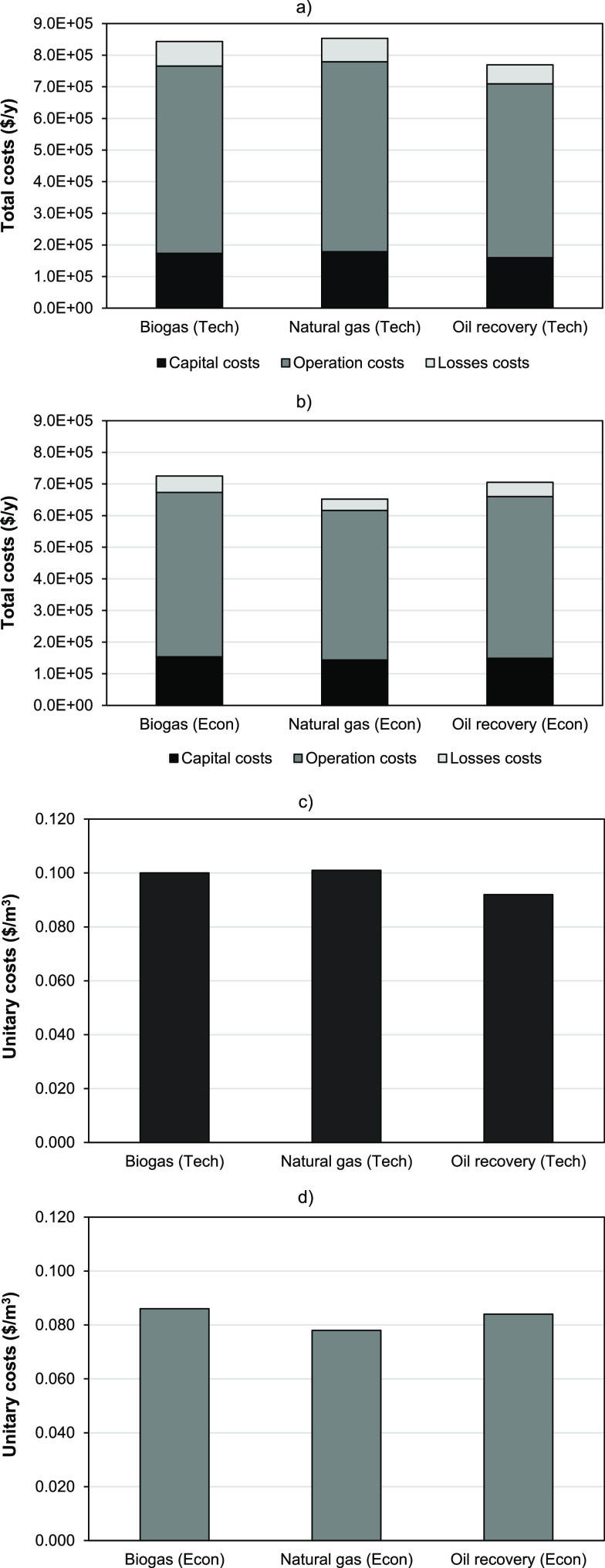
Break-down of the total costs of the process under (a) optimal technical conditions and (b) optimal economic conditions and in terms of unitary costs under (c) optimal technical conditions and (d) optimal economic conditions.

However, the scales of three process are different and cannot be directly compared, so the definition of a common scale equal to 1000 m^3^ STP/h was decided in order to get a clearer comparison. Figure 5 shows the total costs of the processes in this new scale under optimal technical and economic conditions. Within this framework, the natural gas resulted the most expensive case study under optimal technical conditions, but the economic optimization improved greatly its performance (23.6% cost reduction) and resulted the least expensive case under optimal economic conditions. The process for enhanced oil recovery resulted the one with the lowest costs under optimal technical conditions, but the reduction of the costs subject to economic optimization was only 8.4%. In all the case studies, the structure of the costs was similar: the operation costs were the most significant, while the costs due to losses represented the lowest contribution. Among the operation costs, the labor costs and the costs due to membrane replacement after lifetime must be highlighted (around 60 and 30% of the operation costs, respectively), while the utility costs were not so important, with contributions around 10%.

**Figure 6:**
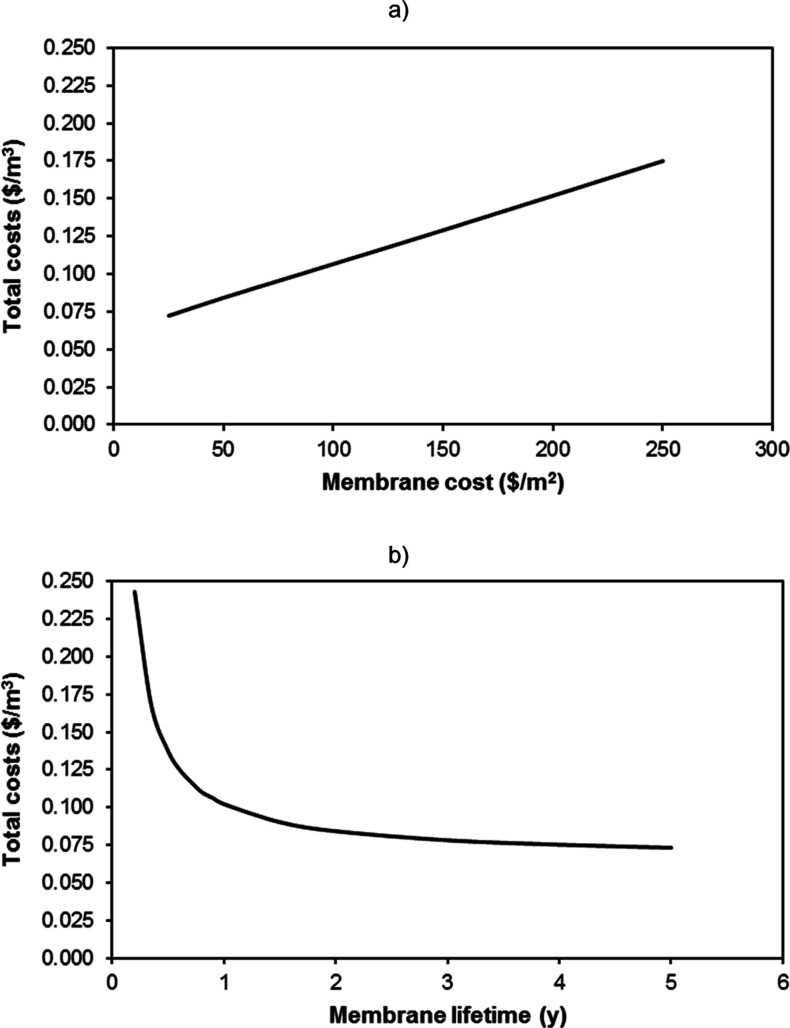
Evolution of the total costs of the oil recovery process as function of (a) membrane cost and (b) membrane effective lifetime (optimal economic conditions in both cases).

To have further information on the sensitivity of the obtained results, different membrane characteristics (cost and effective lifetime) were tested, while the rest of the process variables and parameters in the case of enhanced oil recovery at 1000 m^3^ STP/h feed conditions (Figure 6) were held constant. When the influence of the membrane price was analyzed, a total linear relationship was identified between the total costs and the price of the membrane. The membrane price influenced directly on the capital costs (due to the fixed costs attributable to the membrane modules) and on the operation costs (due to the costs attributable to the membrane replacement after its effective lifetime). This way, a 50% reduction of the membrane price (from 50 to 25 US$/m^2^) implied a reduction of the total costs from 0.084 to 0.072 US$/m^3^ STP (14% reduction), while a 50% increase (to 75 US$/m^2^) augmented the costs to 0.095 US$/m^3^ STP (an equivalent 14% increase). In the case of the membrane effective lifetime, when it decreased below 1 year, the total costs suffered a quick increase, which implied a 63% additional cost when the effective lifetime was reduced to just 6 months (from 0.084 to 0.137 US$/m^3^ STP). Therefore, the stability of the membrane throughout operation time is an essential characteristic that must be deeply investigated for the successful implementation of innovative membranes in order to achieve a competitive scenario. Although the economic assessment of this type of processes depends highly on the method of analysis and assumptions used to evaluate the final results,^110^ this work has demonstrated that the IL2 membrane could be considered a valid alternative for the separation of CH_4_ and CO_2_ mixtures from different sources under competitive conditions; such costs below the margin of 0.050 US$/m^3^ STP identified by other authors could be attained under adequate designs at large scales.^111,112^ This cost range reduces around 50% the estimated cost of biogas upgrading plants using different absorption technologies, such as water scrubbing, pressure swing adsorption, or amine scrubbing.^113^ In fact, even the small-scale installations with feed flowrates below 250 m^3^ STP/h, which resulted in costs around 0.300 US$/m^3^ STP, can be considered within the typical cost interval (0.200–0.400 US$/m^3^ STP) recently defined for standard biogas-upgrading technologies (water scrubbing and commercial membranes) or more advanced options that consider the methanation of captured CO_2_.^102,114^ Further progress in materials engineering and sciences is expected and will further enhance the membrane separation competitiveness for biogas upgrading.^115^

## 6 Conclusions

This work presents the techno-economic optimization of multistage processes for the separation of CO_2_ and CH_4_ mixtures from different sources based on new innovative hollow fiber membranes: a modified commercial polydimethylsiloxane membrane (PDMS_t_) membrane and a non-commercial ionic liquid–chitosan composite membrane (IL2). Three case studies have been covered: biogas upgrading, natural gas sweetening, and enhanced oil recovery.

The optimization results demonstrated that the counter-flow configuration attained the highest technical performance (compared to co-flow and cross-flow configurations), although at the expense of higher membrane area. Only the two-stage processes with both stages containing the IL2 membrane were considered for complete techno-economic optimization of the real scale processes since the designs implementing the PDMS_t_ membrane failed in achieving the purity and recovery restrictions imposed in some of the case studies.

When the technical optimization of the natural gas process was evaluated, the purity of CO_2_ acted as limiting restriction, while the other two case studies were not subject to limiting restrictions (all the imposed purity and recovery values were fulfilled). Therefore, the high initial content of CH_4_ in the feed stream (90% in the case of natural gas sweetening) might imply a great challenge for the separation performance, and only membranes with exceptional selectivity (values above 50 might be suggested) can be implemented for this purpose in order to achieve the required conditions in a two-stage process. Nevertheless, the purity of CH_4_ was the restriction that limited the economic optimization of the process treating biogas and enhanced oil recovery, while in the case of the natural gas, limiting restriction was the recovery of CO_2_.

The scales of the process had a great influence on the total costs, but under equivalent conditions of installation scale, natural gas resulted in the lowest total costs. In all the case studies, the structure of the costs was similar: the operation costs were the most significant, while the costs due to losses represented the lowest contribution. Among the operation costs, the labor costs and the costs due to membrane replacement must be highlighted, while the utility costs were not so important. The sensitivity analyses revealed that the stability of the membrane throughout the operation time is an essential characteristic for the successful implementation of innovative membranes in order to achieve a competitive scenario since short effective lifetimes imply severe economic penalties. Nevertheless, this work has demonstrated that the IL2 membrane, which is based on a biopolymer, could be considered a valid alternative for the separation of CH_4_ and CO_2_ mixtures from different sources under competitive conditions, and costs below the value of 0.050 US$/m^3^ STP could be attained under the adequate design of large-scale installations.

## Supporting Information Available

Parameters of the techno-economic model (PDF)

## Nomenclature

*A*_M_: membrane area of the cell (m^2^)
*A*_mem_: total membrane area of the process (m^2^)
AHE: total area of the heat exchangers (m^2^)
AHE_FC_: area of the heat exchanger of the first stage of the process (m^2^)
AHE_RC_: area of the heat exchanger of the second stage of the process (m^2^)
BPC: base plant costs (US$)
*C*_ELEC_: electricity costs (US$/y)
*C*_LS_: energy loss costs (US$/kJ)
*C*_PFEED_: feed to the first stage heat capacity (kJ/kmol·K)
*C*_PRFEED_: feed to the second stage heat capacity (kJ/kmol·K)
*C*_REF_: refrigeration costs (US$/y)
CC: capital costs (US$/y)
COC: compressors costs (US$/y)
COC_FC_: first stage compressor costs (US$/y)
COC_RC_: second stage compressor costs (US$/y)
DLC: direct labor costs (US$/y)
*F*_in_: total feed flowrate to the module (m^3^ STP/h)
*F*_in2_: total feed flowrate to the module 2 (m^3^ STP/h)
*F*_Ain(*i*)_: flow of compound A in the feed to cell *i* (m^3^ STP/h)
*F*_Bin_: flow of compound B in the feed of the module (m^3^ STP/h)
*F*_in(*i*)_: total feed flowrate to the cell *i* (m^3^ STP/h)
*F*_out_: total retentate flowrate leaving the module (m^3^ STP/h)
*F*_Aout(*i*)_: flow of compound A in the retentate leaving cell *i* (m^3^ STP/h)
*F*_Bout_: flow of compound B in the final retentate of the module (m^3^ STP/h)
*F*_out(*i*)_: total retentate flowrate leaving the cell *i* (m^3^ STP/h)
FC: fixed costs (US$)
FI: facilities investment (US$)
*H*_V_: heating value of lost methane (kJ/m^3^)
HEC: heat exchangers costs (US$)
*I*_GM_: index of the global performance of the module (−)
*I*_GP_: index of the global performance of the process (−)
IC: insurance and taxes costs (US$/y)
ILC: indirect labor costs (US$/y)
*J*_(*i*)_: total gas flowrate through the membrane in cell *i* (m^3^ STP/h)
*J*_A(*i*)_: flow of compound A through the membrane in cell *i* (m^3^ STP/h)
*J*_B(*i*)_: flow of compound B through the membrane in cell *i* (m^3^ STP/h)
LC: total labor costs (US$/y)
LSC: losses costs (US$/y)
MC: maintenance costs (US$/y)
MEC: membrane modules costs (US$)
MRC: membrane replacement costs (US$/y)
*n*_LAB_: number of operators (−)
OC: operation costs (US$/y)
OSF: on-stream factor (−)
*P*_atm_: atmospheric pressure (atm)
*P*_F_: pressure in the retentate lumen side (atm)
*P*_Q_: pressure in the permeate shell side (atm)
*P*_inT_: pressure in the turbine feed (atm)
*P*_outFC_: pressure after the compressor of the first stage (atm)
*P*_outRC_: pressure after the compressor of the second stage (atm)
PC: project contingency costs (US$)
Perm: membrane permeability (GPU)
Perm_A_: permeability of the most permeable compound A (m^3^ STP/h·m^2^·atm)
Perm_B_: permeability of the least permeable compound B (m^3^ STP/h·m^2^·atm)
PI: total project investment (US$)
purity_MA_: purity of the compound A leaving the module (%)
purity_MB_: purity of the compound B leaving the module (%)
purity_PCO_2__: purity of the compound CO_2_ leaving the process (%)
purity_PCH_4__: purity of the compound CH_4_ leaving the process (%)
*Q*_in(*i*)_: total permeate flowrate entering the cell *i* (m^3^ STP/h)
*Q*_Ain(*i*)_: permeate flow of compound A entering the cell *i* (m^3^ STP/h)
*Q*_out_: total permeate flowrate leaving the module (m^3^ STP/h)
*Q*_out1_: total permeate flowrate leaving the module 1 (m^3^ STP/h)
*Q*_out2_: total permeate flowrate leaving the module 2 (m^3^ STP/h)
*Q*_Aout_: flow of compound A in the final permeate of the module (m^3^ STP/h)
*Q*_out(*i*)_: total permeate flowrate leaving the cell *i* (m^3^ STP/h)
*Q*_Aout(*i*)_: permeate flow of compound A leaving the cell *i* (m^3^ STP/h)
*Q*_D_: daily gas flowrate to treat (m^3^/d)
*Q*_FC_: heat exchanged in the compression of the first stage of the process (kW)
*Q*_FEED_: gas flowrate compressed in the first stage (kmol/s)
*Q*_LS_: lost methane flowrate (m^3^/y)
*Q*_RC_: heat exchanged in the compression of the second stage of the process (kW)
*Q*_RFEED_: gas flowrate compressed in the second stage (kmol/s)
*Q*_TFEED_: gas flowrate compressed in the turbine (kmol/s)
*Q*_TREAT_: annual gas flowrate to treat (m^3^/y)
*R*: universal gas constant (kJ/kmol·K)
*r*_PFC_: first compressor pressure ratio (−)
*r*_PRC_: second compressor pressure ratio (−)
recovery_MA_: recovery of the compound A by the module (%)
recovery_MB_: recovery of the compound B by the module (%)
recovery_PCO_2__: recovery of the compound CO_2_ by the process (%)
recovery_PCH_4__: recovery of the compound CH_4_ by the process (%)
SC: start-up costs (US$)
*t*_F_: installation lifetime (y)
*t*_mem_: membrane effective lifetime (y)
*t*_start_: start-up period (y)
*T*_coldFC_: inlet temperature to the first stage compressor (°C)
*T*_coldRC_: inlet temperature to the second stage compressor (°C)
*T*_hotFC_: increased temperature in the first stage compressor (°C)
*T*_hotRC_: increased temperature in the second stage compressor (°C)
*T*_inREF_: inlet temperature of cooling water (°C)
*T*_inT_: inlet temperature to the turbine (°C)
*T*_outREF_: outlet temperature of cooling water (°C)
TC: total costs (US$/m^3^)
TUC: turbine costs (US$)
*U*: overall heat exchanger coefficient (kW/m^2^·K)
UC: utilities costs (US$/y)
WFC: work of the first stage compressor (kW)
WRC: work of the second stage compressor (kW)
WT: work of the turbine (kW)
*x*_A(*i*)_: molar fraction of compound A in the lumen side of cell *i* (−)
*x*_Ain_: molar fraction of compound A in the feed stream to the module (−)
*x*_Ain(*i*)_: molar fraction of compound A in the feed stream to cell *i* (−)
*x*_Aout_: molar fraction of compound A in the final retentate of the module (−)
*x*_B(*i*)_: molar fraction of compound B in the lumen side of cell *i* (−)
*x*_Bin_: molar fraction of compound B in the feed stream to the module (−)
*x*_Bout_: molar fraction of compound B in the final retentate of the module (−)
*x*_CO_2_in2_: molar fraction of compound CO_2_ in the feed to the module 2 (−)
*x*_CH_4_in2_: molar fraction of compound CH_4_ in the feed to the module 2 (−)
*y*_A(*i*)_: molar fraction of compound A in the shell side of cell *i* (−)
*y*_Ain(*i*)_: molar fraction of compound A in the permeate stream entering the cell *i* (−)
*y*_Aout_: molar fraction of compound A in the final permeate of the module (−)
*y*_B(*i*)_: molar fraction of compound B in the shell side of cell *i* (−)
*y*_Bout_: molar fraction of compound B in the final permeate of the module (−)
*y*_CO_2_out1_: molar fraction of compound CO_2_ in the permeate from the module 1 (−)
*y*_CH_4_out1_: molar fraction of compound CH_4_ in the permeate from the module 1 (−)
*y*_CH_4_out2_: molar fraction of compound CH_4_ in the permeate from the module 2 (−)
*Z*_ELEC_: electricity price (US$/kJ)
*Z*_LAB_: labor salary (US$/h)
*Z*_mem_: membrane price (US$/m^2^)
*Z*_REF_: cooling water price (US$/kJ)
α: membrane selectivity (−)
θ: module stage cut (−)
θ_1FC_: temperature difference 1 in the first stage heat exchanger (°C)
θ_1RC_: temperature difference 1 in the second stage heat exchanger (°C)
θ_2FC_: temperature difference 2 in the first stage heat exchanger (°C)
θ_2RC_: temperature difference 2 in the second stage heat exchanger (°C)
Δ*T*_FC_: logarithmic mean temperature difference in the first stage heat exchanger
Δ*T*_RC_: logarithmic mean temperature difference in the second stage heat exchanger
η: isentropic efficiency (−)
γ: ratio *C*_*p*_/*C*_*v*_ (−)
