# Supplementary material for: Techno-Economic Optimization of Multistage Membrane Processes with Innovative Hollow Fiber Modules for the Production of High-Purity CO2 and CH4 from Different Sources
Source: Ind Eng Chem Res. 2022 Jun 2;61(23):8149–65. doi: 10.1021/acs.iecr.2c01138 (PMC9204776; doi:10.1021/acs.iecr.2c01138)
Supplement: Supplementary file 1 — ie2c01138_si_001.pdf [file ie2c01138_si_001.pdf]

TECHNO-ECONOMIC OPTIMIZATION OF MULTISTAGE MEMBRANE PROCESSES WITH INNOVATIVE HOLLOW FIBER MODULES FOR THE PRODUCTION OF HIGH-PURITY CO_2 AND CH_4 FROM DIFFERENT SOURCES

Ricardo Abejón [a *], Clara Casado-Coterillo [b], Aurora Garea [b]

[a] Departamento de Ingeniería Química, Universidad de Santiago de Chile (USACH),
Av. Libertador Bernardo O'Higgins 3363, Estación Central, Santiago 9170019, CHILE
[b] Departamento de Ingenierías Química y Biomolecular, Universidad de Cantabria,
Av. Los Castros s/n, 39005 Santander, SPAIN

* Email: ricardo.abejon@usach.cl

Supporting Information

Table S1. Parameters of the techno-economic model.

Parameter	Unit	Value	References
PDMS CO_2 permeance	GPU	266	1
PDMS selectivity	-	3.1	1
$PDMS_t$ CO_2 permeance	GPU	73.7	1
$PDMS_t$ selectivity	-	10.7	1
IL2 CO_2 permeance	GPU	102	1
IL2 selectivity	-	54.8	1
Heat capacity ratio γ	-	1.351	2
Isentropic efficiency η	-	0.7	2
Reference gas temperature T_{cold}	ºC	30	Own decision
Inlet temperature of cooling water T_{in}	ºC	5	Own decision
Outlet temperature of cooling water T_{out}	ºC	15	Own decision
Overall heat exchanger coefficient U	$kW/m^2 \cdot K$	0.58	2
Specific heat capacity C_{PFEED}, C_{PRFEED}	kJ/kmol·K	36.5	2
On-stream factor OSF	-	0.96	Own decision
Electricity price Z_{elec}	\$/kJ	$0.02 \cdot 10^{-3}$	2
Refrigeration price Z_{ref}	\$/kJ	$4.43 \cdot 10^{-6}$	2
Membrane price Z_{memb}	$\$/m^2$	50	Own decision
Salary Z_{lab}	\$/h	8.5	Own decision
Number of operators N_{lab}	-	2	Own decision
Membrane lifetime T_{memb}	y	2	Own decision
Heating value HV	kJ/m^3	$4.194 \cdot 10^4$	2
Methane loss value CLS	\$/kJ	$20 \cdot 10^{-6}$	3

References

(1) Abejón, R.; Casado-Coterillo, C.; Garea, A. Multiobjective Optimization Based on "Distance-to-Target" Approach of Membrane Units for Separation of CO_2/CH_4. Processes 2021, 9 (11). https://doi.org/10.3390/pr9111871.

(2) Aliaga-Vicente, A.; Caballero, J. A.; Fernández-Torres, J. Synthesis and Optimization of Membrane Cascade for Gas Separation via Mixed-Integer Nonlinear Programming. AIChE J. 2017, 63 (6), 1989–2006. https://doi.org/10.1002/aic.

(3) EUROSTAT. Gas Prices by Type of User. 2021.
